# Limits to Indigenous Participation: The Agta and the Northern Sierra Madre Natural Park, the Philippines

**DOI:** 10.1007/s10745-014-9673-5

**Published:** 2014-07-12

**Authors:** Tessa Minter, Jan van der Ploeg, Maria Pedrablanca, Terry Sunderland, Gerard A. Persoon

**Affiliations:** 1 Leiden University, Leiden, Netherlands; 2 Cagayan Valley Program on Environment and Development (CVPED), Cabagan, Philippines; 3 Center for International Forestry Research (CIFOR), Bogor, Indonesia

**Keywords:** Agta, Hunter-gatherers, Indigenous participation, Protected area management, Philippines

## Abstract

Increased attention for indigenous rights in relation to nature conservation has in the Philippines resulted in legislation formalizing indigenous peoples’ participation in protected area management. We discuss the implementation of this legislation, based on the case of the Agta inhabiting the Northern Sierra Madre Natural Park. The Agta are hunter-gatherers who settle along the coasts and rivers of northeast Luzon. Being indigenous to the park, they hold one third of the seats in its management board. However, our content analysis of this management board’s meetings, combined with qualitative observations of the Agta’s position in the park, show that their participation in its management is hampered by socio-cultural, practical, financial and political barriers. We demonstrate that formalizing indigenous participation in protected area management is not enough to break through existing power structures that inhibit marginalized stakeholders to defense of their interests in natural resources against those of more powerful actors.

## Introduction

The position of indigenous peoples in protected areas is a widely discussed topic in academic and policy debates on conservation and development (Naughton-Treves *et al.* 2005; Agrawal and Redford 2009). The insight that biodiversity rich areas tend to overlap with areas inhabited by indigenous peoples (Kemf 1993; Posey 1999; Gorenflo *et al.* 2012) and criticism of the injustice done to indigenous populations in the context of top-down, centralistic conservation schemes (Colchester 2003; Chapin 2004) have given rise to the notion that conservation of cultural and biological diversity need to be related processes (West *et al.* 2006; Adams and Hutton 2007: 162–3; Pilgrim and Pretty 2010).

Recognition of this relatedness is underpinned in international declarations such as the UN Declaration on the Rights of Indigenous Peoples (UN 2007) and the Convention on Biological Diversity (UN 1992) as well as in policy guidelines by major conservation agencies (IUCN and WWF 2000; WWF 2008). In fact, rights-based approaches to integrating basic human needs and welfare with conservation are articulated and have been adopted by many international organisations (Campese *et al.* 2009; Springer *et al.* 2010).

National governments have responded to these developments in various ways (Persoon *et al.* 2004; Kuper 2003:395). The Philippines have a progressive reputation in this respect. The succession of the Marcos regime in 1986 by a more inclusive administration marked the end of earlier oppressive policies. Rather than being considered illegal squatters on State land, indigenous peoples came to be regarded as partners in protecting what remained of the country’s natural resource base (Vitug 1993; Poffenberger and McGean 1993; Leonen 1998:22; Persoon *et al.* 2004:220; Aquino 2004:62, 64).

**Map 1:**
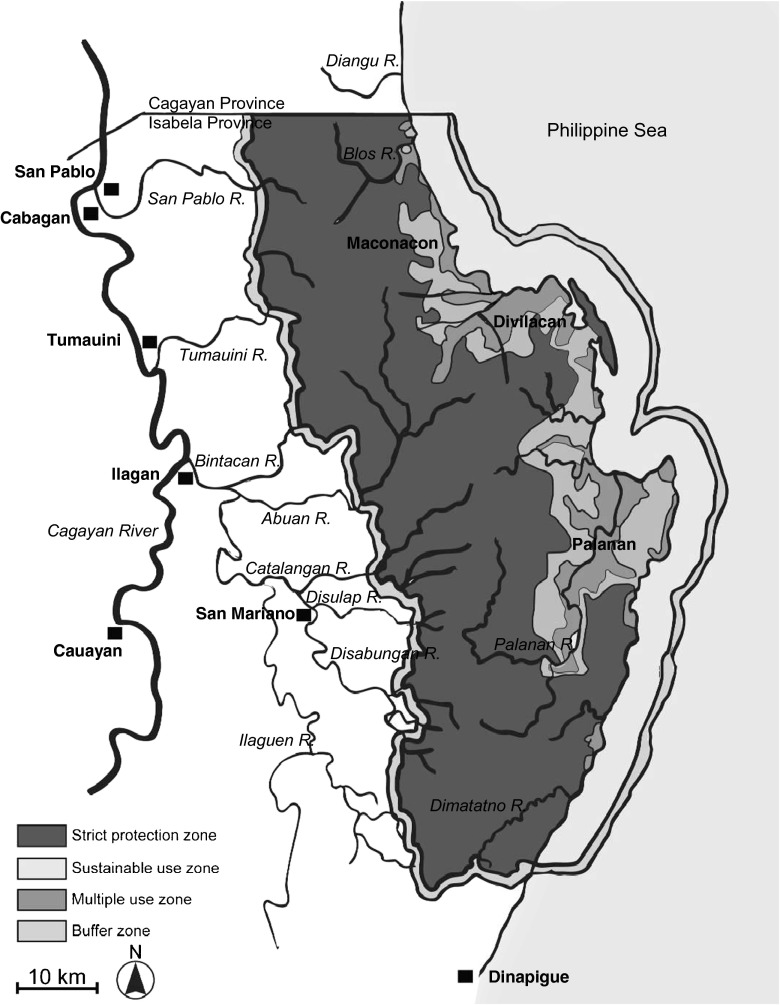
The Northern Sierra Madre Natural Park and its management zones (Minter 2010:28)

Two laws reflect this paradigm shift (Bryant 2000). First, the National Integrated Protected Areas System (NIPAS) Act, seeks to conserve biodiversity through protected areas, but on the condition that indigenous peoples can continue to live and extract resources within park boundaries, and participate in park management (DENR 1992). Second, the Indigenous Peoples’ Rights Act (or RA 8371) allows indigenous peoples to hold collective legal title to their territories, called ‘ancestral domains’ and protects them from displacement by outside activities (NCIP 1997). This paper documents the implications of these legal instruments for the position of the indigenous Agta in the Northern Sierra Madre Natural Park (hereafter NSMNP), the Philippines’ largest protected area (Map 1).

The notion of ‘participation’ gained ground as part of democratization processes that took place throughout the world from the 1960s onwards. The term has become widely used in diverse governance fields including urban planning, tourism development, education and indeed in environmental management. Scholars across these fields of interest have come up with typologies that mostly consider participation to occur along a spectrum from situations where citizens are completely powerless to influence decision making to situations where citizens are ‘in charge’ (e.g. Arnstein 1969; Pretty 1995).

Although such models have been criticized for being overly hierarchical by assuming that some forms of participation are inherently more democratic than others, they still are the best option for assessing how the theoretical idea of participation turns out in practice (Burton 2004:196). This is imperative, for while the concept is widely promoted and its successes are widely claimed, it often is questionable to what extent such claims are valid and whether the outcomes are desirable. In Arnstein’s words:

“There is a critical difference between going through the empty ritual of participation and having the real power needed to affect the outcome of the process. […] [w]ithout redistribution of power [participation] is an empty and frustrating process for the powerless. It allows the powerholders to claim all sides were considered, but makes it possible for only some […] to benefit (Arnstein 1969:216).”

Although participation in the management of protected areas by local people in general, and indigenous people in particular, is frequently required by government programs, the practice of participation has remained remarkably free from empirical scrutiny (Burton 2009:263). In as far as systematic studies assessing participation are conducted at all, they tend to be largely qualitative in nature and lack a methodology that allows for the measurement of participation (Clarke 2008:891–2; Burton 2009:271–274; Lestrelin *et al.* 2011). We aim to contribute to the filling of this gap by asking how often, in what form, by whom and to what effect Agta participation in decision making processes occurs. We thereby seek to provide a more precise understanding of the nature of and limits to indigenous participation in protected area management.

Although the Agta are only one among several stakeholder groups in the park’s natural resources, we focus on their participation in park management for the following reasons. First, subsisting mostly on hunting, fishing and gathering, they depend on the park for sustenance more heavily than local farming populations. Second, possibly because of this distinct lifestyle, at the time of the park’s establishment the Agta were considered to be the main indigenous population of the area. As we will show, this has implications for their right to participate in park management. Finally, the characteristics of hunter-gatherer political organization pose specific challenges to their participation.

## Methods

The results are based on ethnographic research on livelihood strategies of the Agta of the NSMNP that took place between 2002 and 2009 in the context of a doctoral dissertation (see Minter 2010), and yearly follow-up field trips until 2013. Methods used in this study included structured and informal interviewing, qualitative observation, demographic surveys and quantitative studies on time allocation, hunting- and fishing success and agricultural production. In addition, use is made of the results of a project that we initiated in 2006, which aimed to maximize the participation of the Agta in the Protected Area Management Board (hereafter PAMB). Finally, we conducted a content analysis of the minutes of the 39 meetings of this PAMB that took place between 2001 and 2008.

## The Agta and the Northern Sierra Madre Natural Park

Being descendants of the Philippine Archipelago’s first colonizers who settled on the islands between 35,000 and 60,000 years ago (Bellwood 2005), the Agta are recognized as indigenous people under the Indigenous Peoples’ Rights Act (IPRA) (1997). Today, they number around 10,000 individuals, belonging to 16 different language groups (Headland 2010), living throughout the Sierra Madre Mountain Range in Northeast Luzon. The Agta follow a mixed livelihood strategy consisting of fishing, hunting, gathering, barter with neighbouring farmers, logging, paid labour and extensive agriculture. Although some groups are more sedentary than others (Early and Headland 1998), the Agta under study retain considerable mobility. Depending on economic opportunity, as well as spiritual and social needs, Agta shift between various settlement sites. Mobility is however limited by kinship relations: Agta have access to resources only in areas inhabited by relatives. Agta live together in residential groups consisting of between three and fifteen closely related households. Within these residential groups there is no recognized leader, although some elderly people do serve as providers of advice or mediators in conflicts (Headland 1987; Griffin 1996; Minter 2010).

The Agta and their ancestors have maintained socio-economic relationships with non-Agta for at least 4,000 years (Headland and Reid 1989). The number of non-Agta residents in the Northern Sierra Madre has however much increased in the second half of the 20th century, as immigrants came in search of land and employment in the logging industry that was vibrant in Isabela until the early 1990s. As a consequence of immigration, logging and the associated conversion of forest land into agricultural land, pressure on the Agta’s hunting and fishing grounds has correspondingly increased. Racial discrimination, lack of government representation and low educational participation further characterize the Agta’s situation. Finally, Agta society is troubled by an armed conflict between the Armed Forces of the Philippines (AFP) and the New People’s Army (NPA) (the armed wing of the Communist Party) that has been ongoing since the 1970s, and during which the Agta have been recruited and victimized by both parties (Headland and Headland 1997; Early and Headland 1998; van den Top 2003).

Out of the total Agta population of 10,000 people, around 1,800 live within or on the boundaries of the NSMNP in some 80 settlements (Minter 2010). The park was established in 1997 under the National Protected Areas System (NIPAS, 1992).^1^ In 2001 the Congress of the Philippines adopted Republic Act no. 9125, or the Northern Sierra Madre Natural Park Act. In the same year, a park management plan was drafted (Persoon and van Weerd 2006:92).

The NSMNP is the Philippines’ largest protected area, covering nearly 360,000 ha, 75 % of which consists of terrestrial and 25 % of marine habitat (Persoon and van Weerd 2006:93). It covers Luzon’s last undisturbed lowland dipterocarp rainforest, and further includes montane forest, limestone forest, mangroves, beach forest and coral reefs. It also supports a great number of threatened bird, mammal, amphibian, reptile and marine species, many of which are endemic to the Philippines (Mallari *et al.* 2001:154–60).

The NSMNP falls within the provincial boundaries of Isabela and overlaps with nine municipalities. Around 23,000 people live within the protected area’s boundaries, of which less than 8 % are Agta (DENR 2001; Minter 2010). The remainder of the park’s inhabitants consist of various farming populations, most of which are migrants of Ilocano, Ifugao and Tingguian origin who have settled in the Sierra Madre foothills over the past half century. Others, namely the Ibanag, Kalinga and Paranan, have long inhabited the area. As will be explained below, the park’s management plan outlines a zoning system that takes the presence of these human communities into account and prescribes that they are represented in the Protected Area Management Board (DENR 2001:72–81).

The NSMNP is threatened mainly by illegal logging, agricultural expansion, over-hunting and unsustainable fishing practices (DENR 2001:52–4; van der Ploeg *et al.* 2011). Potential future threats come from pending mining claims and a recently approved proposal for the construction of a road across the park.

## The Agta’s Rights and Resource Use in the Park

The NIPAS law stipulates that people who have inhabited or cultivated land in a protected area for at least 5 years prior to its establishment, cannot be relocated against their will. In line with this law, the NSMNP Act and the park management plan provide for a zoning system with respect to settlement and resource use within the park. Areas that were known to be permanently inhabited by farming communities were declared sustainable use zones and multiple use zones (Map 1). Both these zones are open to most forms of resource extraction by all park residents. In contrast, the strict protection zone, which covers over 240,000 ha (67 % of the total protected area), is only open for resource use and settlement by the Agta (see Persoon and van Weerd 2006:93).

Agta are thus granted more extensive resource use and settlement rights within the park than non-Agta. Several arguments underlie this policy. First, the Agta’s indigenous status implies that they cannot be displaced or relocated from a protected area against their will (Indigenous Peoples’ Rights Act, section 58). Second, the earlier mentioned paradigm shift towards a perception of indigenous communities as environmental stewards also applies to the Agta, who are widely assumed to ‘live in harmony with nature’ (e.g. Magaña 2000:1).

The Agta’s resource use rights are limited in two ways. First, the park management plan prescribes that within the strict protection zone the Agta are only allowed to engage in ‘traditional resource utilization’ (DENR 2001:73), but without providing an unambiguous definition of ‘traditional’.^2^ According to the NSMNP Act (DENR 2001a: section 3 l) it refers to resource extraction in which ‘no power machinery’ is used and which is consistent with ‘historically customary techniques of production’. While the former logically implies a prohibition of the use of chainsaws, the latter condition is much less clearly understood. A further limitation is the legal prohibition on exploiting endangered species. This mainly concerns two species of sea turtle, namely the green turtle (*Chelonia mydas*) and the logger head turtle (*Caretta caretta*) (van Lavieren 1999:14, 17). The Agta do not sympathize with prohibitions on turtle exploitation. They claim that, in contrast to non-Agta, who allegedly catch turtles year-round, they exclusively hunt turtles for subsistence purposes during the lean rainy months (see also Magaña 2003:255–6).

Without wishing to trivialize the importance of the above discussion, it must be noted that the park presently has little impact on the Agta’s daily lives. Illustrative in this respect is the fact that despite NGOs’ information campaigns, Agta, and Agta women in particular, are generally badly informed on the park’s existence, its zoning system and the rules and regulations. In a survey of 53 Agta adults, 55 % of respondents were not at all aware of the NSMNP’s existence, while none of the respondents were able to elaborate on the park’s rules and regulations (Minter 2010:254–5).

This limited awareness is at least in part due to weak law enforcement. The NSMNP is a paper park: illegal activities are rarely sanctioned and as a result resource use practices, including those of the Agta, have not been significantly altered by the park’s establishment. From the following overview of their main livelihood activities, it is clear that some of these are compatible with the park’s management plan, while others are not. In our description below, we will distinguish between coast-dwelling and river-dwelling Agta groups as both emphasize different livelihood activities. In all cases however, Agta subsist on a mixed economy, which is based on procuring wild products through fishing, hunting and gathering. They exchange part of these products with non-Agta residents and traders for rice, corn, coffee, sugar and other commodities. In addition, most Agta practice some form of agriculture, which ranges from highly extensive shifting cultivation to semi-permanent rice cultivation. The yields do however nowhere sustain Agta families throughout the year and farming remains just one component of the Agta’s mixed and flexible livelihood package.

### Coast-Dwelling Agta

For coast-dwelling Agta, marine fishing forms the main income generating activity. In the dry season, when the sea is calm, men and women intensively engage in spear fishing. Women do this in pools on top of reef flats, while men usually concentrate on the deeper waters behind the reefs. Several types of self-produced spears, combined with home-made goggles (*anti*-*para*), serve to catch a variety of fish species. In addition, women daily gather octopi, crabs, shellfish and sea cucumbers on reef flats. Nets, and hook and line are occasionally used. Coast-dwelling Agta sell about half of their fishing produce to local buyers, while they consume the remainder.

Commercial lobster fishing has been a major income activity for coast-dwelling Agta for the past two decades. Middlemen selling the lobster on regional markets or in Manila, supply Agta fishermen with lobster traps which are set-up on the reef. Live lobsters are collected from Agta settlements once or twice a week and paid with rice, coffee, sugar and other products. In the wet season, when traps easily get damaged by strong waves, Agta manually collect lobsters from the reef at night. For almost all coast-dwelling Agta lobster fishing generates indispensible income. They are worried however that lobster-stocks will be depleted and have repeatedly urged the PAMB to act against over-fishing.

In the wet season, coast-dwelling Agta turn to other sources of income than fishing alone, as the sea is often turbulent and inaccessible. In these lean months, hunting becomes more important as a livelihood activity. Wild pig (*Sus philippinensis*) and deer (*Rusa marianna*) are the Agta’s main game animals. Agta hunters use bow and arrow, home-produced guns and snare traps, or a combination of these. Especially the use of traps is a contested issue among hunters: while some disapprove of this method for sustainability reasons, others use large numbers of them at once. Competition over game with non-Agta communities is high.

The forest is also sought for several species of rattan, which is sold to local buyers. In recent years, another important non-timber forest product concerns the nests of two species of swiftlet. Breeding in caves, these birds produce nests from saliva that are a highly priced commodity at local, national and international markets. Some Agta men earn a relatively good income from collecting the nests, although this resource too is over-exploited and may soon cease to provide sufficient earnings. Small swiddens, which are planted with root crops and vegetables, provide supplemental sources of food, as do the coconut plantations of non-Agta neighbours from which Agta are usually allowed to harvest. Coast-dwelling Agta occasionally work as land labourers on nearby farms when there is demand for planting or harvesting labour. Their work is paid on a daily basis in kind or cash.

### River-Dwelling Agta

River-dwelling Agta depend on the forest year-round. They live along the forest fringe, and in some cases in the forest interior, usually below elevations of 500 m. Like their coast-dwelling counterparts, they engage in fishing, but the rivers give much lower fishing returns than the sea. Moreover, the use of nets and traps is impractical in the rocky streams, and so spear-fishing is the most important fishing method. Hunting is of much greater importance than along the coast. It is undertaken throughout the year, although more frequently in the wet season.

For river-dwelling Agta the collection of several other forest products for consumption, medicinal use and trade is of great importance. These include honey, fruits, yams, rattan, and swiftlet nests. Another commercially important forest product is timber. In the western interior, Agta settlements serve as gateway to the forest for logging teams. Although many Agta disapprove of logging as it destroys their hunting, fishing and gathering grounds, they lack the power to provide effective resistance against logging. Unable to turn the tide, the majority of river-dwelling Agta men therefore frequently act as tree-pointers, chainsaw operators and log-transporters (Minter and Ranay 2005). As one of these men explains:

‘[…] loggers keep on entering and they are going everywhere. I have told them that they are not allowed to log here, but they asked me: why can’t we log here, was it you who planted the trees? Of course we are not the ones who planted the trees. So we cannot do anything […]’

River-dwelling Agta in upland areas all maintain small swiddens planted with upland rice, root crops, corn and vegetables. These fields are situated on marginal land, and the yields are generally low, although they do provide so-called famine food. As these remote Agta groups generally live at considerable distance from non-Agta farmers, they rarely engage in paid land labour. The situation is different for river-dwelling Agta living in lowland areas, especially on the eastern side of the mountain range. They live in close vicinity to rice farmers to whom they regularly provide labour. Moreover, in recent years, in these areas some Agta have developed their own irrigated rice fields, from which they may harvest up to twice yearly.

## Agta Participation in Park Management

Agta throughout the NSMNP regularly express concern that over-exploitation of wildlife and timber is negatively affecting their livelihood and food security. Interestingly, these sentiments also come from those who are involved in illegal logging (see Minter *et al.* 2005; Minter and Ranay 2005). In theory the park’s management board is the appropriate body to address these matters to. As will be illustrated below, reality is different.

In line with the NIPAS Act (DENR 1992), the NSMNP is governed by a Protected Area Management Board, which was created in 1998. In the PAMB the main stakeholders to the park’s natural resources are represented. The co-management body controls and supervises the Protected Area Superintendent.

There are 36 PAMB members in total, 12 of whom are indigenous representatives. The other members are the Regional Director of the DENR (who also chairs the PAMB), the provincial officer of the DENR, the planning and development officer of the provincial government of Isabela, all mayors of the nine municipalities covered by the park, the two chairpersons of the coastal and western associations of *barangay* (village) captains, three NGOs,^3^ four representatives of People’s Organizations, one representative from women’s organizations, and one representative from youth organizations.

In principle, each year the PAMB meets four times: twice in general assembly form (En Banc meetings), and twice as executive committee. In the latter, one out of nine seats is held by an Agta representative. The office of the Protected Area Superintendent, which falls under the DENR, acts as the secretariat during PAMB meetings.

### Representation

The Agta Executive Committee member was elected by the PAMB itself during the En Banc meeting of September 21, 2001. Soon after his election, however, he was also employed by the National Commission on Indigenous Peoples (NCIP), the government agency that is mandated with implementing the Indigenous Peoples’ Rights Act. Due to institutional conflict and competition between the DENR and the NCIP, this double role was problematic. Nonetheless, the person in question has never been replaced as Executive Committee member.

The Agta En Banc members were not elected, but appointed by the DENR on the basis of their ‘leadership trait, commitment to serve, and familiarization with NSMNP and PAMB matters’ (PLAN 2001:24-5). This selection shows a strong gender and geographical bias. All Agta PAMB representatives are men. Moreover, they only represent populations from Palanan, Divilacan, Maconacon, San Mariano and Dinapigue, while for unclear reasons no representatives were selected from San Pablo, Tumauini and Ilagan (see Map 1). Within the selected municipalities PAMB representatives are also unevenly distributed. For instance, all three representatives coming from San Mariano are members of two residential groups that are situated in adjacent watersheds.

### Participation

In addition to these representation problems, there are participation problems. Comprising nearly one-third of all board members, the Agta representatives theoretically form a powerful block within the PAMB. In reality, however, their leverage remains weak. This qualitative observation is confirmed by our analysis of the minutes of 39 PAMB meetings that took place from 2001 to 2008. Of these, 22 were Executive Committee meetings and 17 were En Banc meetings.

Agta attendance was most problematic at En Banc meetings. On average, these meetings were attended by only four out of twelve Agta members. It is important to note that this figure is shaped partly by two particular meetings which had full Agta attendance because they were co-organized by us in 2007 and 2008, as will be further discussed below. Without this, Agta attendance would have been much lower. The situation was better at the meetings of the Executive Committee: the only Agta member of this body was present at 80 % of the 22 meetings.

Participation, however, is not synonymous to presence. It is therefore useful to look at the frequency with and manner in which Agta PAMB members contribute to the discussions taking place in these meetings. As Table 1 shows, the minutes of the 39 PAMB meetings studied contain 60 individual cases in which topics were discussed that are directly relevant to the Agta. Several observations can be made from looking at these discussions.

**Table 1:** Discussions on topics relevant to the Agta during 39 PAMB meetings (2001–2008)

	Frequency	%
Discussions on topics relevant to the Agta (total)	60	100
With Agta participation in the discussion	26	43
Active interpellations by Agta	18	
Rattan gathering permits	4	
Illegal logging	3	
Overfishing (mainly lobster)	3	
Ancestral domains	3	
Request for financial support for travel to PAMB meetings	2	
Others	3	
Passive responses by Agta	8	
Without Agta participation in the discussion	34	57
Ancestral domains	11	
Livelihood and/or empowerment projects	9	
Rattan permits	6	
Presentations on Agta related research	3	
Mining operations	2	
Others	3	

First, in as much as 57 % of the cases, Agta matters were discussed without any involvement of Agta PAMB members themselves. One third of these concern heated and lengthy debates on the NCIP’s proposed declaration of the entire NSMNP as Agta ancestral domain. Although this controversial issue is of enormous importance to the Agta’s future (see Minter 2010:258–263), the Agta PAMB members, including above-mentioned NCIP employee, are strikingly absent from discussions about it (see Table 1 for details on other topics).^4^

In the remaining 43 % of the cases the Agta did take part in the discussion. In 8 of these cases (31 %) their role was limited to answering a question directed at them. Agta PAMB members intervened in the discussion 18 times (69 %). Most of these interpellations were expressions of concern over unsustainable resource extraction in the NSMNP. As the example below illustrates, however, some more powerful PAMB members tend to avoid action in relation to these concerns. The minutes of the PAMB En Banc meeting of August 6, 2008, contain the following discussion on an initiative to stop illegal logging:

‘[An Agta PAMB member] mentioned that the said issue should not be delayed […]. He also added that PAMB already conducted lots of meetings and made plans but actions are very slowly and the Northern Sierra Madre is really in threat condition so it needs to be rescued. […] He asked the cooperation of all PAMB members to have stronger power to fight the illegal logging problems inside the Sierra Madre; to save the remaining forest. The Acting Chair [Mayor X] replied that there was no invitation for the PAMB […]. Mayor [Y] informed the body that it is inappropriate for them to decide rather to defer a motion for the formation of a new task force, considering that there are legal issues that should be raised and threshout [sic]; like the mandate of each organization representing each other; and in terms of confiscation as to which organization is mandated to do so […]’

### Practical Barriers to Participation

Thus, not only are PAMB meetings poorly attended by Agta members, in as far as Agta members are present, they hardly influence the agenda and decision making. There are a number of reasons for this. First, the Agta’s physical presence at PAMB meetings is hampered by the fact that announcements for meetings and notifications on changes in the schedule often do not reach them on time, if at all. Second, no financial mechanism is in place to support the Agta’s travel to and from meetings. This is despite the fact that the NSMNP Act (DENR 2001a: section 11) states that PAMB members are entitled to compensation for travelling and subsistence expenses and should receive a honorarium and insurance coverage whenever they attend PAMB meetings. Third, the Agta’s socio-economic disadvantages restrain their active participation. In spite of the agreement that Tagalog or Ilocano are to be spoken during meetings, English, which is not understood by most Agta, is often used. In addition, the Agta’s non-literacy inhibits their understanding of the minutes, the agenda and any other written documents that provide a background to the meeting’s process and contents. Finally, being unfamiliar with the aim, setting and structure of meetings like these, Agta PAMB members are unable to use them to their advantage.

### Facilitating Participation

The Agta face a situation in which they are not given a realistic chance to actively participate in park management. In 2006, this observation led us to initiate a small project which aimed to maximize participation of Agta members in the PAMB of the NSMNP in order to strengthen their voting power. Various activities were developed, the most important of which will be summarized here.

To tackle the practical and logistical problems underlying the Agta’s poor participation in the PAMB, the project lobbied with municipal governments and the DENR for structural funding of travel costs to and from meetings, as well as a system which ensures that invitations are being sent out well in advance of meetings. To address the Agta’s poor understanding of the protected area and the PAMB, two-day community consultations were held among the residential groups of all Agta PAMB members; and two trainings were organized for the Agta PAMB members. These trainings were well-attended: on both occasions all Agta PAMB members were present.

In the course of the project duration (2006–2008) hardly any lasting impacts were generated with respect to the practical and logistical problems. Although lobbying activities did result in promises by almost all municipalities to make funding available, only one municipality actually formalized this promise in a resolution. In addition, the invitation system did not significantly improve, and thus continued to pose a problem to Agta PAMB members’ attendance.

With respect to the Agta’s empowerment several achievements were made. During the community consultations, Agta expressed satisfaction with their improved understanding of the NSMNP and the PAMB, and actively contributed issues to be listed on the PAMB agenda through their PAMB representatives. These mostly concerned appeals to stop illegal logging, as well as destructive forms of hunting and fishing in their living areas. Also, Agta PAMB members felt empowered by the trainings that they attended and were hopeful that they could make use of their acquired knowledge and skills during PAMB meetings. Agta PAMB member attendance in meetings significantly increased during the project cycle as compared to the period prior to the project.

However, although the Agta’s attendance at PAMB meetings did increase, their involvement in discussions taking place during these meetings hardly did. Agta PAMB members’ proposed agenda items were not included in the meeting’s main agenda, even if this list was submitted to the secretariat well in advance of the meeting. Moreover, if they did speak up, the issues discussed were often dominated by other stakeholders. Also, the issues they raised were not always reflected in the minutes of the meeting.

## Discussion: Limits to Participation

In his typology of participation, Pretty (1995:1252) outlines a continuum from what he calls ‘manipulative participation’ to ‘self-mobilization’. The Agta’s formal position in the NSMNP as stipulated under the NIPAS Act, the IPRA and the NSMNP Act, suggests they would come under one of the more empowered categories of the continuum, which is characterized by joint decision making processes, joint design of management plans and the view that participation is a right. However, in practice the Agta’s case is indeed exemplary of ‘manipulative participation’, in which case ‘Participation is simply a pretence, with “people’s” representatives on official boards but who are unelected and have no power’ (Pretty 1995:1252).

The case presented shows that this lack of ‘meaningful participation’ (Clarke 2008) is partly due to practical problems regarding communication, finances and logistics. Also, hunter-gatherers’ participation in park management appears particularly problematic because they form the least empowered, least organized, least understood and, probably therefore, least respected indigenous populations in any country. Management plans designed towards hunter-gatherers’ improved participation are especially prone to cultural and economic insensitivities. For example, the procedure which resulted in the Agta PAMB members’ selection is not compatible with the Agta’s socio-political organization. As mentioned, although certain elderly Agta men and women are highly respected and may have a role as advisor or even mediator, there is no such thing as a recognized leader.

However, the most fundamental limitation to meaningful participation is formed by existing power structures: patronage networks often inhibit Agta PAMB members to publically raise concerns regarding natural resource management. An underlying problem is that the protected area itself contradicts the vested economic interests and development agenda of regional elites, and their allies in local government (Utting 2000). This clarifies to a large extent why the concerns of the Agta are often deliberately countered, delayed or evaded in PAMB meetings. The situation is exemplary of a political system that is closed to participants who want to modify institutions in response to negative ecological feedback (Alcorn *et al.* 2003:300).

At the same time, Agta PAMB members are pushed into a role of substitute park guards. Both government and non-government organizations regularly turn to the Agta for help in environmental protection. However, although the PAMB is authorized to deputize individuals for enforcement of rules and regulations within the protected area through the Protected Area Superintendent (DENR 2001a: section 10f), Agta are often pushed to stand up against illegal activities without such formal deputization. This means that while they confront trespassers, they lack the authority to do so.

Also, while the IPRA states that indigenous communities “shall be given the responsibility to maintain, develop, protect and conserve [protected areas]”, it also prescribes that this should be done “with the full and effective assistance of government agencies” (NCIP 1997: section 58). However, examples abound in which Agta’s reports on illegal activities to village governments, municipal mayors, the DENR, or police are never followed up. As the following quote from an Agta PAMB member illustrates, the greatest frustration arises from this lack of support:

‘Our role is to report illegal activities like illegal logging and electro-fishing. […] I want to request the DENR officials to come here one day to monitor the area. We as PAMB members here cannot control the illegal loggers. Or else, they will shoot us.’

## Conclusion

The increasing recognition of indigenous peoples’ rights in relation to natural resource management in general and conservation efforts in particular has in the Philippines resulted in the formalization of their role in protected area management. The representation of indigenous communities in park management boards is legally required and consistently integrated in park management plans. While these measures provide the institutional basis for according indigenous people an explicit role in park management, we have shown that they do by no means warrant their ‘meaningful’ participation.

Our systematic measurement of the indigenous Agta’s participation in the management of the Philippines’ largest protected area, the NSMNP, confirms our qualitative observation that their role is marginal. An analysis of the minutes of 39 meetings of the PAMB in the period 2001–2008 shows that the Agta board-members are rarely present during these meetings. Moreover, even when they do attend their ability to influence the meetings’ agenda and outcomes is minimal.

When taking a close look at what participation means on the ground it becomes clear that its efficacy is limited by practical, financial, socio-cultural and political factors. The most fundamental barrier is posed by existing power structures, which ensure that the interests of dominant stakeholders to natural resources override those of less powerful actors. Participation even risks becoming a pretext for government agencies to put the weight of their own responsibilities on indigenous shoulders. By continuously emphasizing the Agta’s role as environmental stewards, government burdens them with a task for which they are not equipped and not compensated, while it masks the lack of political will to enforce the law.

Under these circumstances, efforts at maximizing participation, as the project that was described in this paper aimed for, merely results in increased frustration among the indigenous people involved. To get back to Arnstein (1969:216) once more: as long as no redistribution of power takes place, participation processes are empty rituals which maintain the status quo.

## Footnotes

1 This was an extension of the Palanan Wilderness Area which was declared by President Ferdinand Marcos in 1979.

2 Note that under the NSMNP Act (DENR 2001a: section 3jj), the Agta’s resource rights in the strict protection zone are even much more narrowly defined as resource extraction for ‘ceremonial and religious use’.

3 Conservation International, WWF-Philippines, and the Mabuwaya Foundation (an NGO working on community based conservation of the Philippine crocodile).

4 The only reasonably consistent form of participation of the Agta membership is the Executive Committee member’s frequent leading of the opening prayer: he was asked to do so at over 20 % of all meetings. This is remarkable as most Agta adhere an animistic belief system.
